# Modifications of Hippocampal Circuits and Early Disruption of Adult Neurogenesis in the Tg2576 Mouse Model of Alzheimer’s Disease

**DOI:** 10.1371/journal.pone.0076497

**Published:** 2013-09-27

**Authors:** Alice Krezymon, Kevin Richetin, Hélène Halley, Laurent Roybon, Jean-Michel Lassalle, Bernard Francès, Laure Verret, Claire Rampon

**Affiliations:** 1 Université de Toulouse (UPS) Centre de Recherches sur la Cognition Animale, Toulouse, France; 2 Centre National de la Recherche Scientifique (CNRS) Centre de Recherches sur la Cognition Animale, Toulouse, France; 3 Multi Park, Department of Experimental Medical Science, Wallenberg Neuroscience Center, Lund University, Lund, Sweden; University of South Florida, United States of America

## Abstract

At advanced stages of Alzheimer’s disease, cognitive dysfunction is accompanied by severe alterations of hippocampal circuits that may largely underlie memory impairments. However, it is likely that anatomical remodeling in the hippocampus may start long before any cognitive alteration is detected. Using the well-described Tg2576 mouse model of Alzheimer’s disease that develops progressive age-dependent amyloidosis and cognitive deficits, we examined whether specific stages of the disease were associated with the expression of anatomical markers of hippocampal dysfunction. We found that these mice develop a complex pattern of changes in their dentate gyrus with aging. Those include aberrant expression of neuropeptide Y and reduced levels of calbindin, reflecting a profound remodeling of inhibitory and excitatory circuits in the dentate gyrus. Preceding these changes, we identified severe alterations of adult hippocampal neurogenesis in Tg2576 mice. We gathered converging data in Tg2576 mice at young age, indicating impaired maturation of new neurons that may compromise their functional integration into hippocampal circuits. Thus, disruption of adult hippocampal neurogenesis occurred before network remodeling in this mouse model and therefore may account as an early event in the etiology of Alzheimer’s pathology. Ultimately, both events may constitute key components of hippocampal dysfunction and associated cognitive deficits occurring in Alzheimer’s disease.

## Introduction

Alzheimer’s disease (AD) is a progressive neurodegenerative disease characterized by deep impairments of learning and memory processes. These deficits develop along with the occurrence of main histological hallmarks such as senile plaques composed of β-amyloid peptides (Aβ), neurofibrillary tangles formed of hyperphosphorylated tau, progressive synaptic and neuronal loss in specific brain regions. The hippocampus is one of the primary targets of these anatomical alterations and is also a key player in the formation of declarative memory. Although behavioral assays remain the ultimate readout of brain dysfunction in the disease, more convenient representative measures of hippocampal deficits have been sought for the past years. As it is the case in patients, amyloid plaque load does not correlate with cognitive performance in mouse models of AD [1–5]. Recently, other markers of hippocampal functional modifications reflecting cerebral dysfunction have been described in mouse models of AD [6–9]. Among them, aberrant expression of the neuromodulator neuropeptide Y (NPY) and depletion of the activity-dependent protein calbindin D-28k (CB) are thought to reflect hippocampal modifications induced by Aβ/human Amyloid Precursor Protein (hAPP) toxicity and pathogenicity [6,7,9–11]. In several mouse models of AD, the expression of some of these functional markers was shown to correlate with each other and with the severity of learning and memory impairments [6,7,10]. It was hypothesized that these modifications of the hippocampal circuits may actively contribute to the observed behavioral deficits associated with AD pathology [12]. Beside the alteration of the aforementioned remodeling of inhibitory and excitatory circuits, abnormal adult hippocampal neurogenesis might also contribute to the early symptoms of AD, including the inability to store new information [13,14]. Although the exact contribution of hippocampal newborn neurons to learning and memory processes remains debated, it was consistently reported that new neurons in the adult brain contribute to cognitive processes related to hippocampal function [15,16]. Hippocampal neurogenesis was studied in a number of mouse models of AD that display amyloid deposition [14,17]. Overall, these reports reveal that hippocampal neurogenesis is altered to an extent that may differ depending on the transgenic line that is used and the stage of AD at the time of study. Previous reports on hippocampal modifications in mice focused on models depicting aggressive progression of amyloid pathology, exhibiting dramatic increase of Aβ production and accelerated onset of amyloid plaques formation, like APPxPS1dE9 mice [11,18,19], hAPPJ20 mice [6–8,20] or APPxPS1Ki mice [21,22]. Oddly, little is known about the evolution of these markers in the Tg2576 mice, whilst it is one of the most studied models of the disease with regards to amyloid pathology and cognitive deficits. Tg2576 transgenic mice overexpress the human APP isoform harboring the Swedish double mutation [23]. At young age (<5 months) these mice show increasing levels of soluble Aβ [3,24,25] and by 6-8 months of age, they display high levels of both Aβ40 and Aβ42 in the brain [23]. Amyloid plaques start forming in the hippocampus and cerebral cortex of Tg2576 mice at 9-12 months of age and continue to increase thereafter to levels observed in AD brains [23,26], while CSF levels of Aβ42, but not Aβ40, decrease with age and amyloid deposition [26]. Concomitantly, Tg2576 mice develop age-related memory deficits that are well characterized in numerous behavioral tasks and paradigms at different stages along the disease progression [5,23,27–31]. Undoubtedly, the course of the pathology in Tg2576 mice, is more reminiscent of the findings in AD patients than other mouse models mimicking more aggressive forms of amyloid pathology, and thus should provide new insights on the extent to which aging and amyloidogenesis may relate to hippocampal dysfunction. To address this issue, we evaluated expression of markers associated with aberrant hippocampal remodeling (NPY and CB) and we characterized adult hippocampal neurogenesis at specific stages along the progression of the pathology in Tg2576 mice.

## Material and Methods

### Ethics statement

All experiments were performed in strict accordance with the recommendations of The European Communities Council Directive (86/609/EEC), The French National Committee (87/848) and the guide for the Care and Use of Laboratory Animals of the National Institutes of Health (NIH publication nu 85–23). In application of the European directive 2010/63/UE and according to the ongoing French legislation at the time of experiments, no specific approval was necessary for this study. However, the Centre de Recherches sur la Cognition Animale (CRCA) has received French legal approval for experiments on living vertebrate animals (Arrêté Préfectoral dated 9-02-2011) and this work was carried out in accordance with the Policies of the French Committee of Ethics. Animal surgery and experimentation conducted in this study were authorized by the French Direction of Veterinary Services to LV (#31–238, 2005) and CR (#31-11555521, 2002) and all efforts were made to improve animals’ welfare and minimize animals suffering.

### Transgenic mice generation and genotyping

Male mice from the transgenic line Tg2576 created by Hsiao et al. [23,32], were used for these experiments. Tg2576 mice carry a double mutation (Lys670-Asn, Met671-Leu [K670N, M671L]), driven by a hamster prion protein promoter, and over-express human APP695. Tg2576 males were bred with C57B6/SJL F1 females (Charles River, L’Arbresle, France) and the offspring were genotyped to confirm the presence of human APP DNA sequence by PCR [23,32]. All experiments were conducted in males to avoid the impact of estrus cycle onto neurogenesis process. Transgenic mice (Tg2576) and age-matched non-transgenic male littermates (NTg) were maintained on a 12hrs light/12hrs dark cycle with free access to food and water and were used at the age of 3, 5, 12 or 18 months.

### BrdU injection

To assess levels of hippocampal cell proliferation, 3, 5 or 12 months Tg2576 and NTg mice received two intraperitoneal injections two hours apart, of 5-bromo-2-deoxyuridine (BrdU) (100 mg/kg in 0.9% saline; Sigma, St. Louis, MO). Twenty-four hours later, mice were deeply anaesthetized and transcardially perfused with 0.1 M phosphate buffer (PB; pH 7.4) followed by 4% paraformaldehyde. To assess new cell survival, additional groups of mice received three injections of BrdU (100 mg/kg) four hours apart and were perfused 30 days later, as described above.

### Injection of the retroviral vector

The Moloneyleukemia-derived retroviral vector pCMMP-IRES2eGFP-WPRE expressing enhanced green fluorescent protein (MoMulV::GFP) [33] was used to study the morphology of adult-born neurons in the hippocampus of Tg2576 and NTg mice. This vector targets cells that are actively replicating at the time of infection, such as neural progenitors cells located in the neurogenic zones of the central nervous system [33]. Mice were anesthetized (ketamine 120mg/kg, xylazine 10mg/kg) and the viral solution was infused into the dentate gyrus bilaterally (0.5 ml of MoMulV::GFP; coordinates relative to bregma : anteroposterior -2 mm; lateral ±1.6 mm; ventral -2.5 mm). Mice were perfused 14 or 28 days later, as described above.

### Tissue processing and immunolabeling

After perfusion, the brains were removed, postfixed overnight in 4% paraformaldehyde solution and transferred for at least 2 days in 0.1M PB containing 30% sucrose at 4°C. Brains were then rapidly frozen in -50°C isopentane. Coronal 30µm-thick sections (or 50µm-thick for GFP staining) were obtained with a cryostat and stored in cryoprotectant at -20°C until use. For immunochemistry, all steps were done at room temperature. Free-floating brain sections were rinsed in PB containing 0.9% NaCl and 0.25% Triton X-100(PBST) before quenching endogenous peroxidases with 3% H_2_O_2_ in 10% methanol in PBS. For BrdU immunochemistry, sections were incubated in 2N HCl for 40 min to denature DNA and then neutralized in 0.1M borate buffer (pH 8.5) before being processed as follow. All the sections were blocked in PBST containing 5% normal goat serum for 60 minutes, followed by overnight incubation in one of the following primary antibodies: rabbit anti-CB-D28k (1:40,000; Swant, Bellinzona, Switzerland); rabbit anti-NPY (1:5000; Sigma Aldrich, St Louis, MO, USA); monoclonal rat anti-BrdU (1:400; OBT-0030, Harlan Seralab, Loughborough, UK) or rabbit anti-GFP (1:1000; Torrey Pines Biolabs, Houston, TX, USA) in PBST with 0.1% sodium azide (PBST-Az) containing 5% normal goat serum. For GFP fluorescent labeling only, sections were incubated the next day with Alexa 488 donkey anti-rabbit (1:500; Invitrogen, Carlsbad, CA) in PBST before being rinsed and mounted onto slides, coverslipped using Mowiol and stored at 4°C. For CB, NPY or BrdU labelings, sections were incubated for 90 min in biotinylated goat anti-rabbit or biotinylated goat anti-rat antisera (1:400; Vector Laboratories, Burlingame, CA, USA) in PBST. After rinses, sections were then incubated for 90 minutes in avidin-biotin-peroxidase complex (1:500; Vector Laboratories Elite Kit) in PBST. Peroxidase immunolabeling was developed in 0.05 M Tris-HCl buffer (pH 7.6) containing 0.025% 3,3’-diaminobenzidine-4 HCl (DAB; Fluka, Buchs, Switzerland) and 0.003% H_2_O_2_. NPY and BrdU stainings were revealed in black by adding 0.06% nickel ammonium sulfate to the developing solution. The reaction was stopped by rinses in PBST-Az. Sections were mounted onto slides, counterstained with Nuclear Fast Red (Vector Laboratories) for BrdU only, dehydrated through alcohols and coverslipped.

Sections used for triple-immunofluorescent labeling of BrdU with markers of mature (NeuN) and immature (Doublecortin, DCX) neurons were processed for antigen retrieval (10mM sodium citrate buffer, pH6.0, for 30min at 95°C) before DNA denaturation, as described above. They were then incubated for 48h at 4°C in a mix of rat anti-BrdU (1:400, OBT-0030; Harlan Seralab), mouse anti-NeuN (1:500; MAB377, Millipore) and goat anti-DCX antisera (1:300; C-18, Santa Cruz Biotechnology, Santa Cruz, CA, USA) in PBST-Az. After several rinses in PBST, sections were incubated for 90 min at room temperature in a mix of secondary reagents: Alexa 488-conjugated highly cross-adsorbed donkey anti-rat (1:500; Invitrogen), Alexa 647-conjugated donkey anti-mouse (1:500; Invitrogen), and Alexa 555 donkey anti-goat (1:500; Invitrogen) in PBST. Sections were rinsed intensively and mounted onto slides, coverslipped using Mowiol and stored at 4°C.

### Quantification of CB and NPY labeling

All slides were coded prior to analysis so that the experimenter was blind to groups and genotypes until all samples were counted. CB is expressed in dentate granule cells and is visible in both the cell bodies and their dendrites located in the molecular layer. NPY is expressed in a subset of inhibitory interneurons. NPY staining is visible in the soma of these cells, and a faint staining from the axons of these inhibitory interneurons is visible in the molecular layer, where they form synapses onto the dendrites of granule cells. Quantifications were performed as previously described [31]. Briefly, CB and NPY labelings were quantified for each animal by measuring immunoreactivity density in the molecular layer and mossy fibers on two serial coronal sections spaced at 360µm through the dorsal hippocampus. The CA1 stratum radiatum and the latero-posterior thalamic nucleus were used as internal controls to normalize for variations in signal intensity for CB and NPY, respectively. Digitized images were obtained with a digital camera (Optronics, Goleta, CA) on a light microscope (x10 objective, BX51 Olympus, Essex, UK) using the Mercator Pro stereology system (Explora Nova, La Rochelle, France). Integrated optical density (IOD) was measured using image analysis software ImageJ (open source, available on http://rsbweb.nih.gov/ij/index.html).

### Quantification of BrdU-labeled cells

Quantification of BrdU-immunoreactive (BrdU+) cells was conducted as previously described [15,19]. One in 6 series of sections spaced 180µm and spanning the rostro-caudal extent of the dorsal dentate gyrus was labeled for BrdU. Cells located within the granule cell layer and adjacent subgranular zone, defined as a two cell-body-wide zone along the border between the granule cell layer and the hilus, were counted through a x40 objective. BrdU+ nuclei intersecting the uppermost focal plane were excluded from the count in order to avoid oversampling. The corresponding surface area of GCL/SGZ sampled for counting was measured using the Mercator stereology system (Explora Nova, La Rochelle, France). The reference volume was determined as the sum of the traced areas multiplied by the distance between sampled sections (180µm). Density of BrdU+ cells was then calculated by dividing the number of labeled cells by the granule cell layer sectional volume. The total number of BrdU+ cells was estimated by multiplying these densities by the reference volume.

### Quantification of BrdU/DCX/NeuN triple-labeled cells

The relative number of mature and immature neurons among the surviving BrdU-labeled cell population was measured in mice sacrificed 30 days after BrdU injections. One in 12 series of sections spaced 360µm and spanning the rostrocaudal extent of the dorsal hippocampus was triple labeled for BrdU, NeuN and DCX. Twenty BrdU+ cells in the dorsal dentate gyrus from each animal were randomly chosen for analysis. Co-expression of NeuN or DCX was assessed for each BrdU+ cell, using a confocal laser-scanning microscope (TCS SP2, Leica, Heidelberg, Germany). The analysis was carried on over the entire z-axis using a x100 oil-immersion objective, in a sequential scanning mode to prevent crossover between channels. The mean number of cells for each phenotype was calculated by multiplying the average fraction for each phenotype by the individual BrdU+ cell count obtained in DAB for each animal. Optical images for figures were obtained using Leica confocal software.

### Migration analysis of GFP+ cells

The localization and morphology of green fluorescent protein-expressing (GFP+) cells in the dentate gyrus (DG) of mice were analyzed 14 or 28 days post-injection (dpi) of a retroviral vector encoding GFP. Series of 1 in 3 of 50µm thick coronal sections were processed for GFP immunochemistry and counterstained with Hoechst. Sections surrounding the injection site and displaying tissue damage were discarded. A total of 15–20 sections from at least four mice were used for each time-point. GFP+ cells in the DG were identified as neuron-like cells when presenting a round soma, a conspicuous axonal projection extending toward the hilus and spiny dendrites reaching the outer molecular layer [34]. To evaluate radial migration of GFP+ neuron-like cells, the granular cell layer was virtually divided into inner, middle and outer layers and each GFP+ cell was assigned to one of these layers, the subgranular zone (SGZ) or the hilus.

### Dendritic morphology analysis of GFP+ cells

At 14 and 28dpi, 6-7 cells/mouse were analyzed by acquisition of z-series of 50-75 optical sections at 0.5 µm intervals, with a 40x oil lens, digital zoom of 1.7, with a TCS SP5 (Leica Microsystem) confocal system. Three-dimensional reconstructions of GFP+ granule cells with largely intact dendritic trees were analyzed using Imaris XT (Bitplane, Zurich, Switzerland). Total dendritic length, primary dendrite length, maximal dendritic length and number of branching were calculated automatically from three-dimensional reconstruction. Growth of GFP+ axonal fibers was measured using Mercator software (Explora Nova), according to a method previously described [35].

### Dendritic spine density and spine shape analysis

Dendritic analysis of GFP+ neurons located after the second branching point was performed by acquiring z-series of 30–50 optical sections at 0.2 µm intervals, with a 63x oil lens, digital zoom of 5, with a TCS SP5 (Leica Microsystem) confocal system. Before analysis, files were subjected to seven iterations of deconvolution with the Huygens Essential deconvolution software (SVI, Hilversum, Netherlands). Confocal images were imported into Imaris (Bitplane AG, Zurich, Switzerland) and for analysis. Drift correction, particle tracking, and surface tracking were done using autoregressive tracking algorithms. Dendritic spines were defined as protrusions from the dendritic shaft and classified based on their shape according to Harris et al. [36]. Spines were categorized into four types: filopodia (protrusion with long neck and no head), thin (protrusion with a neck and head <0.6 µm in diameter), stubby (protrusion with no obvious neck or head) or mushroom (protrusion with a neck and a head with a diameter >0.6 µm). Dendritic spine analysis included spine density (number of spines/10micrometers) and morphological classification. For NTg and Tg2576, 10 dendritic segments (corresponding to 200–300 µm length) were analyzed from 3 mice.

### Statistical analysis

Age and genotype interactions were evaluated by two-way ANOVA for multiple comparisons using GraphPad software (Prism, version 4). When a significant difference was found with ANOVA, Bonferroni test was used for post-hoc analysis to identify significant pair wise differences. Homoscedasticity of the data was verified before any statistical analysis using Bartlett test. When comparing two groups, unpaired two-tailed Student t test was used. Results were expressed as mean ± SEM. On graphs, we used *, **, *** for genotype comparisons and #, ##, # # for age comparisons, when the p-values were p <0.05, p <0.01 or p <0.001, respectively.

## Results

### Ectopic expression of the inhibitory neuropeptide Y in the dentate gyrus of Tg2576 mice with aging

Recent data have suggested that high levels of Aβ in the AD brain may destabilize hippocampal network activity, leading to the remodeling of neuronal hippocampal circuits, which in turn, may contribute to memory impairments observed in AD [8,9,37]. These modifications of hippocampal circuits include the development of compensatory inhibitory neuronal networks aimed to suppress aberrant excitatory neuronal activity. Supporting this idea, a profound remodeling of neuronal hippocampal circuits that includes ectopic expression of the inhibitory NPY has been identified in several mouse models of AD [6,8,11,38], including 13 month-old female Tg2576 mice [31]. We examined here whether such abnormal expression of NPY may occur in Tg2576 mice as a function of their age and severity of the disease. NPY expression was thus quantified in the DG of Tg2576 mice at 3, 5, 12 and 18 months of age, corresponding to milestones in the development of the disease in this mouse model [26].

**Figure 1:**
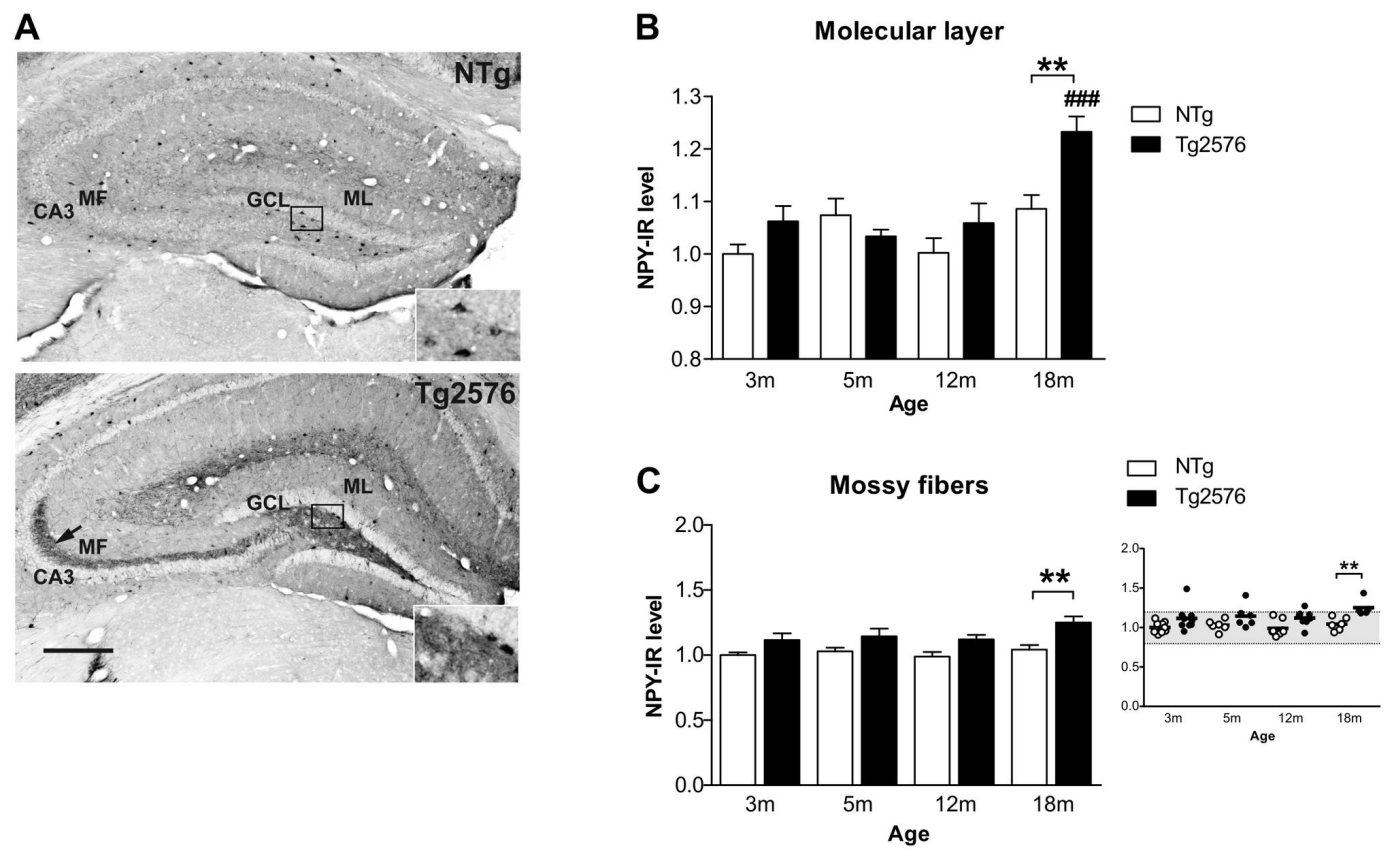
Ectopic expression of inhibitory neuropeptide Y in the dentate gyrus of Tg2576 mice with aging. (A) Neuropeptide Y (NPY) immunoreactivity in the dentate gyrus of 18 months-old NTg and Tg2576 mice. Albeit NPY staining was observed in the soma of some hilar cells in both NTg and Tg2576 mice (enlargements), a massive ectopic NPY expression in the mossy fibers running from the hilus to CA3 (arrow) was evidenced in Tg2576 mice only. (B) NPY immunoreactivity (NPY-IR) was quantified in the molecular layer of NTg and Tg2576 mice as a function of age (in months) (n=5-10 mice/group). NPY expression remained stable through aging in NTg mice while it increased significantly in Tg2576 mice at 18 months compared to 3, 5 and 12 month-old Tg2576 mice and compared to age-matched NTg control mice. (C) Quantification of NPY expression in the mossy fibers of Tg2576 and NTg mice along aging. An ectopic expression of NPY was observed in the mossy fibers of several Tg2576 mice, in proportions that increased with age, becoming statistically significant at 18 months, as shown in the scattered plot where each dot represents a mouse. Quantitative data represent mean ± SEM in B and C. Data were analyzed by two-way ANOVA with Bonferroni post-hoc: ###p<0.001 age effect for each genotype; **p<0.01 Tg2576 mice *vs* age-matched NTg, two-way ANOVA. ML: molecular layer, GCL: granular layer, MF: mossy fibers, CA3: Ammon’s horn 3. Scale bar: A=300µm.

In the molecular layer of the DG, NPY expression remained stable across aging in NTg mice but increased significantly in 18 month-old Tg2576 mice compared to younger Tg2576 mice (transgene effect: F(1,50)=7.7, p=0.007; p<0.001, when compared to 3, 5 and 12 month-old Tg2576 mice with ANOVA followed by Bonferroni post-hoc) and compared to age-matched NTg control mice (p<0.01) (Figure 1A,B). These data are consistent with our recent report showing no change in NPY expression in the molecular layer of 13 month-old female Tg2576 mice [31] and thus indicate that sprouting of NPY immunoreactive fibers may occur between 13 and 18 months of age in these mice.

Aberrant expression of NPY in the mossy fibers has often been associated with epileptic activity in the hippocampus or with the occurrence of a generalized seizure in the days before sacrifice [8,11,39]. In the present work, an overall increased ectopic NPY expression was observed in Tg2576 mice (genotype effect F(1,50)=24.5; p<0.001) and post-hoc analysis revealed that this effect reached significance at 18 months compared to age-matched NTg mice (p<0.01, Bonferroni post-hoc) (Figure 1C). Interestingly, analysis of NPY immunoreactivity pattern at the individual level revealed that the proportion of Tg2576 animals that displayed a strong ectopic NPY labeling in their mossy fibers increased with age (3m: 1/9, 5m: 1/6, 12m: 1/8, 18m: 3/5) (Figure 1A,C). Consistent with these data, we previously described that a substantial proportion of 13 month-old Tg2576 female mice was showing aberrant expression of NPY in their mossy fibers [31]. Moreover, NPY labeling in the mossy fibers across aging, was positively correlated with NPY ectopic expression in the molecular layer (r of pearson: 0.73, p<0.0001, data not illustrated). Importantly, none of the NTg mice displayed this aberrant NPY expression.

### Reduction of the activity-related protein CB expression in the dentate gyrus of Tg2576 mice with aging

Using other mouse models of AD, previous studies have shown that remodeling of the neuronal hippocampal circuits is also characterized by a depletion of synaptic activity-related proteins that may in turn contribute to the cognitive decline associated to AD. In particular, depletion of calcium-binding protein CB can be observed in both the soma (granular cell layer) and dendritic processes (molecular layer) of granule cells in mouse models depicting an aggressive progression of the disease [6,7,10,11,38,40,41].

**Figure 2:**
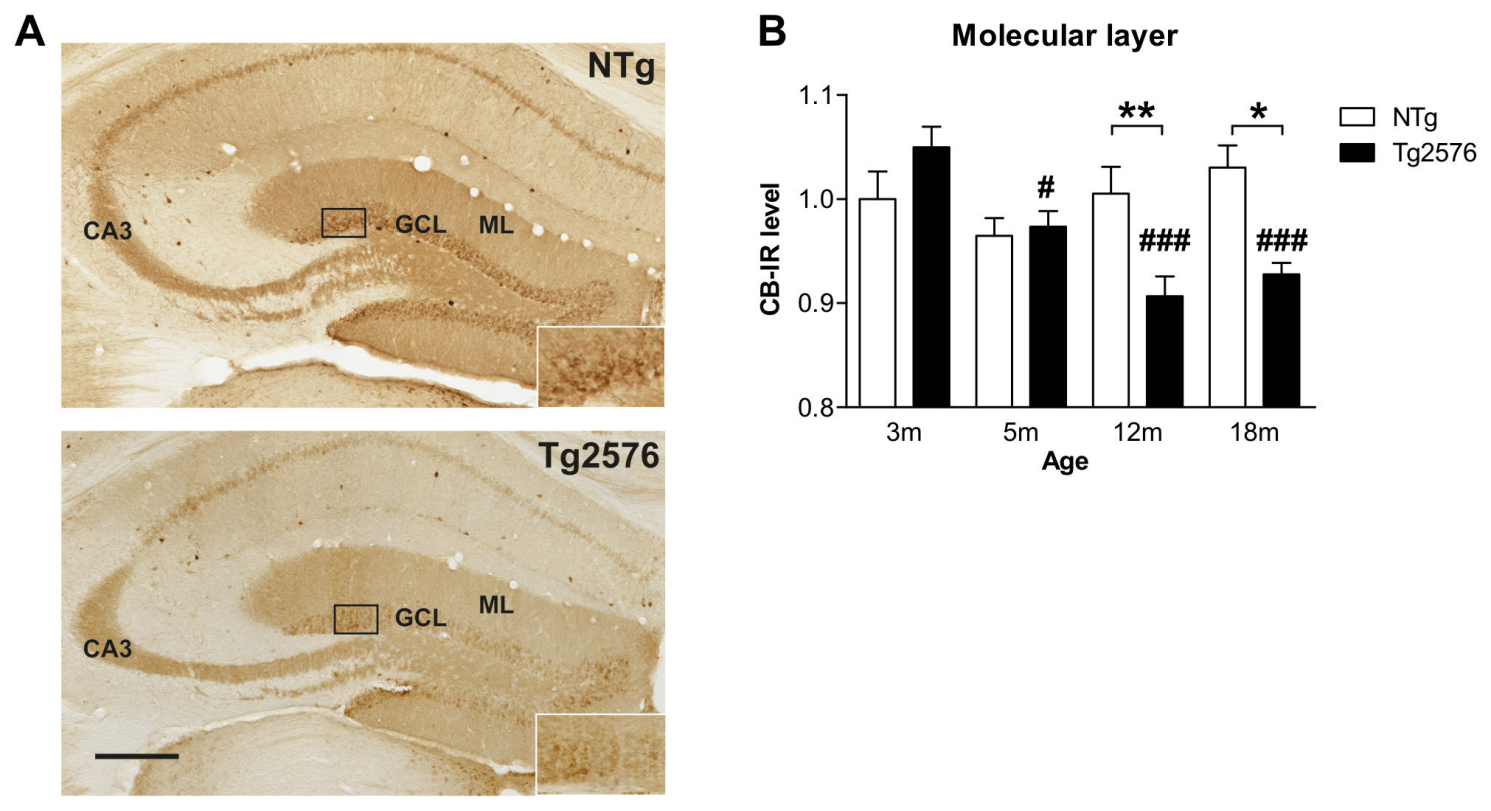
Late depletion of calbindin expression in dentate gyrus of Tg2576 mice. (A) Brain sections of 12 month-old NTg and Tg2576 mice immunolabeled for calbindin (CB). (B) CB expression was evaluated in the molecular layer of the dentate gyrus and remained stable through the aging process in NTg mice (n=6-9 mice/group). In Tg2576 mice, CB expression decreased gradually with aging starting at 5 months compared to 3 month-old Tg2576. Compared to age-matched NTg mice, 12 and 18 month-old Tg2576 mice displayed strongly reduced CB expression. Quantitative data represent mean ± SEM in B. Data were analyzed by two-way ANOVA with Bonferroni post-hoc: #p<0.05; ###p<0.001 age effect for each genotype; *p<0.05; **p<0.01 Tg2576 mice *vs* age-matched NTg, two-way ANOVA. ML: molecular layer, GCL: granular layer. Scale bar: A=250µm.

Analysis of CB expression in the molecular layer of the DG revealed a significant effect of genotype (F(1,51)=5.5; p=0.02) and age (F(3,51)=5.5; p=0.006), as well as an age x genotype interaction (F(3,51)=6.8; p=0.0006, two-way ANOVA) (Figure 2A,B). Whilst CB expression remained stable in aging control mice, it decreased gradually with age in Tg2576 mice (Figure 2A, B). Eventually, 12 and 18 month-old Tg2576 mice displayed significantly reduced levels of CB in the molecular layer compared to age-matched NTg mice (Figure 2B). However, these data obtained in males differ from our recent report showing no change of CB expression in the molecular cell layer of 13 month-old female Tg2576 mice [31].

Altogether, these findings reveal that Tg2576 display anatomical alterations in the DG that are likely to disturb hippocampal neuronal activity and plasticity in aging Tg2576 mice. The DG is also a neurogenic niche where new cells are continuously generated during adulthood and contribute to hippocampal plasticity and memory processes [42]. The maturation and functional integration of these adult-generated cells are sensitive to extrinsic signals available in the local environment [43], suggesting that aberrant remodeling of hippocampal circuits occurring during the course of AD may impinge on adult neurogenesis [20]. We next tested this possibility in Tg2576 mice.

### Early alterations of adult hippocampal neurogenesis in Tg2576 mice

**Figure 3:**
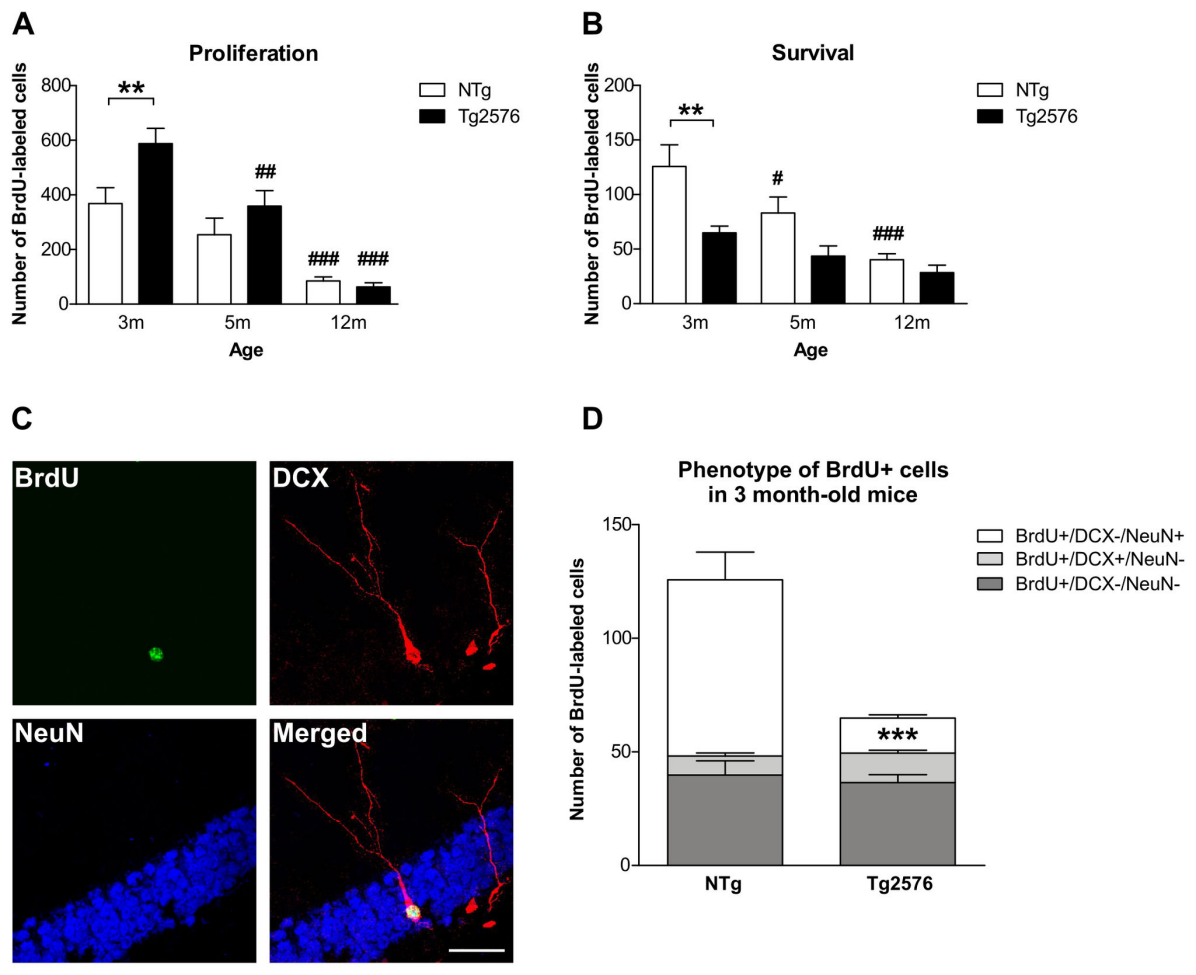
Altered adult hippocampal neurogenesis in 3 month-old Tg2576 mice. (A) New cell proliferation was measured in the dentate gyrus of NTg and Tg2576 mice at different ages (n=8-11 mice/group). Mice from both genotypes exhibit overall decreased numbers of BrdU-labeled (BrdU+) cells in the dentate gyrus along aging, reflecting age-related reduction in cell proliferation. At 3 months, Tg2576 display enhanced proliferative activity compared to age-matched NTg mice. (B) BrdU was injected in 3 month-old mice and numbers of surviving BrdU+ cells were evaluated 30 days after (n=7-9 mice/group). These numbers decreased with age in both genotypes. Moreover, the number surviving BrdU+ cell was drastically reduced in Tg2576 mice injected at 3 months of age compared to age-matched NTg. (C) Confocal analysis was used to score the co-expression of doublecortin (DCX, red) and NeuN (blue) in 30 day-old BrdU+ cells (green) NTg and Tg2576 mice injected at 3 months of age (n=7-9 mice/group). The merged image depicts a BrdU+/DCX+/NeuN- immature neuron. (D) Distribution of BrdU+ cells among cell phenotypes, for both genotypes. Compared to NTg, a significantly smaller number of BrdU+ cells co-labeled with the neuronal marker NeuN in Tg2576 mice. Quantitative data represent mean ± SEM in A, B and D. Data were analyzed by two-way ANOVA with Bonferroni post-hoc: #p<0.05; ##p<0.01; ###p<0.001 age effect for each genotype; **p<0.01; ***p<0.001 Tg2576 *vs* age-matched NTg. Scale bar C=40µm.

We quantified adult hippocampal neurogenesis in Tg2576 and NTg mice aged 3, 5 and 12 months. Because hippocampal neurogenesis declines drastically with age [44], 18 month-old mice were not included in our study. As expected, the number of proliferating BrdU-labeled (BrdU+) cells considerably decreased with age in the DG of both NTg and Tg2576 mice (p<0.001, F(2,48)=44.1; two-way ANOVA) (Figure 3A). Interestingly, although 3 month-old Tg2576 mice exhibited significantly more proliferating BrdU+ cells than age-matched control NTg animals (NTg: 368.6±57.8 *vs* Tg2576: 587.5±56.2 BrdU+ cells/DG; p<0.01, Bonferroni post-hoc), 5 and 12 month-old Tg2576 and NTg mice displayed similar proliferative activity in the DG (Figure 3A). Thus, the overall age-related detrimental effect on the number of proliferating cells was more pronounced in Tg2576 mice than in NTg animals.

New cell survival was assessed 30 days after cell birth. Again, the number of surviving 30 days-old BrdU+ cells in the DG decreased noticeably with age in both genotypes (F(2,44)=14.1; p<0.001) (Figure 3B). The number of surviving BrdU+ cells was strongly reduced in 3 month-old Tg2576 mice compared to age-matched NTg (3m NTg: 125.7±19.9 *vs* Tg2576: 64.9±6.2 BrdU+ cells/DG, p<0.01 Bonferroni post-hoc), while no differences were observed between genotypes at older ages (Figure 3B). Thus, despite enhanced cell proliferation in 3 month-old Tg2576 mice, most of these new cells do not survive over 30 days, revealing a deeply altered hippocampal neurogenesis at an age when Tg2576 mice are still devoid of amyloid extracellular deposits and present no major cognitive impairment.

### Reduced numbers of mature new neurons in the hippocampus of young Tg2576 mice

To further characterize which cell types are missing among the surviving hippocampal new cells of 3 month-old Tg2576 mice, we evaluated the maturational stage of each surviving BrdU+ cell in the DG. Triple fluorescent immunolabeling against BrdU and markers of mature (NeuN) and immature (DCX) neurons was analyzed by confocal microscopy (Figure 3C). Doublecortin (DCX) is a microtubule-stabilizing factor expressed early during neuronal differentiation which expression is down-regulated concomitantly with the appearance of the neuron-specific nuclear protein (NeuN) [45]. To examine whether neuronal maturation of new cells is impacted by the presence of hAPP in Tg2576 mice, we quantified the fractions of surviving BrdU+ cells that coexpressed DCX, NeuN or neither marker (Table 1, left columns). In 3 month-old Tg2576 mice, we found that four weeks after cell division, a significantly smaller proportion of BrdU+ cells expressed the mature neuronal marker NeuN compared to age-matched NTg (transgene effect : F(1,13)=6.5 p=0.01, p<0.01, Bonferroni post-hoc). Consequently, over 50% of the BrdU+ cell population expressed none of the neuronal markers in 3 month-old Tg2576 mice. The impact of hAPP on new neurons fate was the strongest at young age as no significant changes were observed in the proportions of BrdU+ cells expressing NeuN, DCX or neither marker in 5 and 12 month-old Tg2576 mice compared to age-matched controls (Table 1, left columns). One possible explanation for this is that the maturation of adult-born granule cells may be delayed in the brain of young Tg2576 mice.

**Table 1:** Phenotypic distribution of BrdU-immunoreactive cells in the dentate gyrus 30 days after BrdU injection.

		**Fraction of BrdU+ cells expressing**			**Mean number of cells**		
Age at BrdU injection	Genotype	Neither marker (%)	DCX (%)	NeuN (%)	BrdU+/DCX-/NeuN-	BrdU+/DCX+/NeuN-	BrdU+/DCX-/NeuN+
3m	NTg	31.7±6.7	6.7±1.7	61.6±8.3	39.8±6.3	8.4±1.3	77.5±12.2
	Tg2576	56.3±10.3	20.0±7.4	23.7±3.8**	36.5±3.5	13.0±1.2	15.4±1.5***
5m	NTg	43.3±15.9	36.7±24.5	20.0±10.4#	36.0±6.4	30.4±5.4###	16.6±2.9###
	Tg2576	53.1±11.0	12.4±5.1	34.4±11.5	23.2±4.9	5.4±1.1	15.0±3.2
12m	NTg	65.0±12.6	19.2±9.6	15.8±7.4##	26.1±3.6	7.7±1.1	6.4±0.9###
	Tg2576	66.7±3.3	18.3±4.4	15.0±2.9	18.9±4.6#	5.2±1.3#	4.2±1.0

BrdU-positive (BrdU+) cells were phenotyped by colabeling with markers specific for postmitotic neurons (NeuN) or immature neuronal precursors (DCX). The percentage of each phenotype as a fraction of the total BrdU population (left columns) and the corresponding mean number of cells are indicated (right columns). Analysis of percentage of colabeled BrdU+ cells allows us to determine whether the neuronal maturation process is altered with aging and/or with the APP transgene (left column), without taking into account potential alteration of the survival rate. Thus, mean numbers of immature and mature BrdU-positive neurons (right column) were also estimated by multiplying the fraction of BrdU+ cells from each phenotype, by the total number of surviving BrdU+ cells. Data represent mean ± SEM. Significance values indicated for individual pairs are as follows: **p<0.01 ***p<0.001 vs age-matched NTg, #p<0.05 ##p<0.01 ###p<0.001 age effect for each genotype (ANOVA with Bonferroni’s post hoc).

However, because significantly fewer BrdU+ cells survived 30 days in 3 month-old Tg2576 mice compared to age-matched NTg animals (as reported in Figure 3B), we further expressed the triple-labeling quantification data in a way that takes into consideration the difference in new cell survival among mice genotypes. Thus, we calculated for each individual, the corresponding mean number of BrdU+ cells expressing NeuN, DCX or neither marker. Data are expressed as group mean, for each age and genotype in the Table 1 (right columns). Regarding 3 month-old mice, we found that the number of BrdU+ cells expressing a mature neuronal phenotype (BrdU ^+^ /DCX^-^/NeuN^+^ cells) was noticeably diminished in Tg2576 mice compared to age-matched NTg (transgene effect: F(1,44)=26.7 p<0.001; p<0.001, ANOVA, Bonferroni post-hoc) (Figure 3D, Table 1). In contrast, numbers of BrdU+ cells that expressed an immature neuronal phenotype (BrdU ^+^ /DCX ^+^ /NeuN^-^ cells) or neither phenotypic marker (BrdU ^+^ /DCX^-^/NeuN^-^ cells) were not significantly different between genotypes (Figure 3D, Table 1). Hence, the massive reduction of new neurons being added to the DG during adulthood could contribute to the progressive hippocampal dysfunction seen in this AD mouse model. This result also reveals that altered production of new granule cells occurs early in the course of the disease.

### Migration of adult-born hippocampal neurons

**Figure 4:**
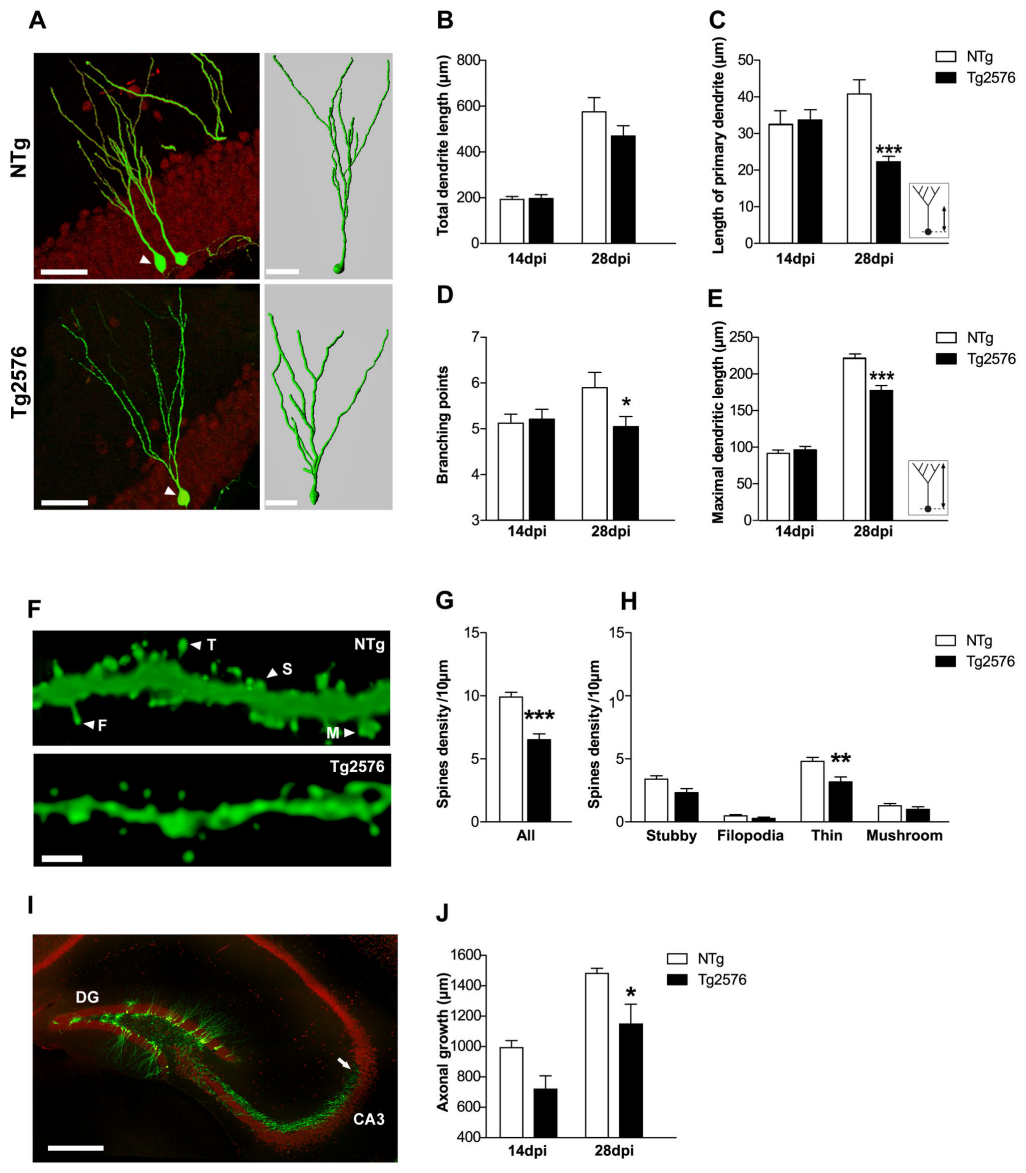
Impaired morphological development of new granule neurons in 3 month-old Tg2576 mice. (A) Confocal images (left) illustrating typical retrovirally GFP-labeled (GFP+, green) neurons 28 days post-injection (dpi) in the dentate gyrus of 3 month-old NTg and Tg2576 mice (neuronal marker NeuN, red) and their corresponding 3D reconstruction (right) illustrating the arborization and branching points of GFP+ neurons. (B) No difference was found in the total dendritic length of GFP+ neurons from NTg and Tg2576 mice (n=3-6 mice/group and 15-20 GFP+ cells/group). (C) The length of the primary dendrite was measured as indicated in inset. At 28dpi GFP+ neurons in Tg2576 mice exhibited a shorter primary dendrite compared to NTg mice (n=3-4 mice/group, n=20-25 GFP+ cells/group). (D) Number of branching points was counted in 14 and 28dpi GFP+ neurons (n=3-4 mice/group, 20-25 GFP+ cells/group). At 28dpi, GFP+ neurons exhibited less branching points in Tg2576 mice compared to NTg mice. (E) Maximal dendritic length of GFP+ neurons as quantified as illustrated in inset. At 28dpi, Tg2576 mice displayed shorter maximal dendritic length compared to NTg (n=3-4 mice/group and n=20-25 GFP+ cells/group). (F) Maximal projection of z-stacks confocal images illustrating dendritic segments from 28dpi GFP+ neurons, from which dendritic spines were counted and classified as stubby (S), filopodia (F), thin (T) or mushroom (M) according to morphological criteria described in the methods. (G) Spine density estimated from 28dpi GFP+ neurons (n=3 mice/group, 4-8 segments/mouse, 200-300µm of dendritic segments analyzed/mouse) was reduced in 3 month-old Tg2576 mice compared to NTg. (H) Among all classes, the density of thin spines was reduced in Tg2576 mice compared to NTg. (I) Confocal image depicting mature neurons (NeuN, red) and 28 day-old new neurons (GFP, green) in the dentate gyrus. Axons of GFP+ neurons (arrow) extending into the CA3 region are clearly visible, allowing the measurement of axonal length. (J) At 28dpi, axons of GFP+ neurons appeared shorter in Tg2576 compared to NTg mice. Data in B, C, D, E, G, H, J represent mean ± SEM. Data were analyzed by two-way ANOVA with Bonferroni post-hoc: *p<0.05; ***p<0.001 Tg2576 *vs* NTg. Scale bars: A (left)=50µm, (right)=30µm F=2µm, I=500µm.

Maturation and functional integration of adult-born granule cells are known to be sensitive to extrinsic signals from the local hippocampal environment [46]. Their proper positioning within the granular cell layer is crucial for adult-generated neurons to adopt mature morphological features and integrate the network. To examine new cell migration and morphology in 3 month-old Tg2576 mice, we used a retroviral vector encoding enhanced green fluorescent protein (GFP) to label adult-generated hippocampal cells (Figure 4A). Migration of new neurons was evaluated by counting the number of GFP-labeled (GFP+) neurons located within the DG (see methods). Analyses were carried out 14 and 28 days post-injection (dpi) of the viral vector corresponding to critical stages of new neurons maturation [35]. Migration of GFP+ neurons within the granular cell layer did not vary between genotypes at any post-injection time-point (Table 2), with about 75% of GFP+ cells localized within the inner third. The remaining GFP+ cells were found in the subgranular zone, where the progenitors lie, or had reached the middle third of the granular cell layer in proportions that were not different across genotypes. Very few GFP+ neurons were positioned in the outer third at these delays (<4%). These data indicate that hAPP/Aβ overexpression in Tg2576 mice does not affect the positioning nor the relative distribution of newborn cells within the layers of the dentate gyrus. Thus, the reduced number of mature newly born neurons in Tg2576 mice could not be attributable to improper migration of these cells within the granular cell layers.

**Table 2:** Localization of adult-born GFP-labeled neurons in the dentate gyrus of young mice.

		**Localization of GFP+ neurons (in %)**			
Days post-injection	Genotype	SGZ	GCL inner	GCL middle	GCL outer
14dpi	NTg	9.6±3.6	86.1±4.0	4.0±1.0	0.3±0.3
	Tg2576	8.5±4.7	74.8±6.9	16.7±11.4	0±0.0
28dpi	NTg	2.8±1.4	74.1±8.9	19.5±7.7	3.6±2.3
	Tg2576	9.7±6.4	74.2±3.6	13.7±6.1	2.1±1.2

Distribution of GFP+ neurons (in %) in the dentate gyrus of mice injected with the retroviral vector at 3 months of age and sacrificed either 14 or 28 days post-injection (dpi). Data represent mean ± SEM. SGZ: subgranular zone; GCL: granular cell layer of the dentate gyrus.

### Morphological maturation of adult-born hippocampal neurons

Morphological development of newborn cells is another critical parameter for their functional integration into hippocampal circuits and consequently, their survival [16,47]. We thus analyzed dendritic arborization and axonal growth of 14 and 28 days-old GFP+ cells harboring neuron-like anatomical features, in 3 month-old Tg2576 and NTg mice (Figure 4). Total dendritic length of GFP+ neurons, that can be used as a rough index of neuronal morphology, was similar in both genotypes at 14dpi (NTg: 193.2±12µm *vs* Tg2576: 196.4±16.3µm, p>0.05, Bonferroni post-hoc) but seemed to be reduced in Tg2576 mice at 28dpi, although it remained under significance (NTg: 575.4±62 µm *vs* Tg2576: 469.5± 45.1 µm, p>0.05, Bonferroni post-hoc) (Figure 4B). To further characterize the potential changes that may occur in new neurons, we evaluated dendritic complexity of GFP+ neurons from their 3D reconstructions (Figure 4A). Thus, we measured the length of the primary dendritic branch of GFP+ neurons, which corresponds to the distance between the soma and the first dendritic branching point. Although similar across genotypes at 14dpi, it was shorter in Tg2576 compared to NTg mice at 28dpi (NTg: 40.9±4.4µm *vs* Tg2576: 21.7±2.0µm, p<0.001, Bonferroni post-hoc) (Figure 4C). The number of branching points on GFP+ neurons at 14dpi was the same regardless of mice genotype (NTg: 4.2±0.4 *vs* Tg2576: 4.4±0,4µm, p>0.05, Bonferroni post-hoc), but was significantly reduced in Tg2576 mice at 28dpi (NTg: 5.8±0.7 *vs* Tg2576: 4.1±0.4, p<0.05, Bonferroni post-hoc) (Figure 4D). The maximal dendritic length of GFP+ neurons, representing the distance from the soma to its farthest neuritic extension, was similar in both genotypes at 14dpi (Figure 4E) (NTg: 91.3±2.8µm *vs* Tg2576: 92.4±4.1µm, p>0.05, Bonferroni post-hoc). However, at 28dpi, maximal dendritic length was shorter in Tg2576 compared to NTg mice (NTg: 219.7±4.5µm *vs* Tg2576: 175.5±6.7µm, p<0.001 Bonferroni post-hoc). Altogether, these observations are indicative of a reduced dendritic development of adult-born neurons in Tg2576 animals compared to controls. Consequently, new granule neurons in Tg2576 mice may not receive the totality of the perforant path inputs from the entorhinal cortex. Because dendritic spines are the predominant synaptic sites of granule neurons to receive excitatory inputs, spine density and shape also reflect integration of newly born neurons into the neuronal network. Adult-generated neurons start forming dendritic spines at 16dpi [35]. We therefore quantified the density of dendritic spines on GFP+ neurons at 28dpi and observed significantly less spines on Tg2576 mice dendrites than in age-matched control mice (NTg: 9.9±0.37 *vs* Tg2576: 6.5±0.46 spines/10µm, p<0.001, unpaired t test) (Figure 4F,G). These findings, in line with our data on dendritic arborization, suggest that new neurons maturation is delayed in 3 month-old Tg2576 mice. Moreover, reduced spine density may reflect impaired connectivity of new neurons in Tg2576 mice. To examine this further, we classified the spines into four morphological types based on their morphology: stubby, filopodia, thin and mushroom (see methods) [48,49]. We found a significant reduction of thin spines density on 28dpi GFP+ neurons in Tg2576 mice (Figure 4H) (NTg: 4.8±0.32 *vs* Tg2576: 3.2±0.40 thin spines/10µm; p<0.001, Bonferroni post-hoc). Densities of stubby, filopodia and mushroom spines present on 28dpi GFP+ neurons seemed to decrease in Tg2576 mice, although it did not reach significance (Figure 4H). Thus, reduced spine density of new neurons in Tg2576 mice appears to be merely related to changes in the density of labile, immature spines rather than to changes in the population of stabilized mushroom spines (Figure 4H). Thus, 28 days after their birth, new neurons in Tg2576 mice hold a reduced potential for synaptic integration compared to new neurons in control mice.

Finally, axonal growth of new neurons was estimated by measuring axonal projections of 14 and 28dpi GFP+ cells towards the CA3 region in 3 month-old mice (Figure 4I). In both genotypes, axonal fibers grew significantly between 14 and 28dpi (time effect: F(1,14)=36.8; p<0.001) (Figure 4J). However, GFP+ axonal fibers were significantly shorter in Tg2576 compared to NTg at 28dpi (NTg: 1484±34.7µm *vs* Tg2576: 1150.8±130.2µm, p<0.05, Bonferroni post-hoc) (Figure 4J). As a result, axons of GFP+ cells in control mice reach a farther area into CA3 at 28dpi compared to Tg2576 mice. Taken together, these data reveal that the morphological development of new granule neurons is impaired in Tg2576 mice and as a consequence, their functional integration into hippocampal circuitry may be compromised.

## Discussion

This study revealed that Tg2576 mice, an AD mouse model expressing the hAPP form carrying the Swedish mutation develop a complex pattern of changes in the dentate gyrus of their hippocampus that appears with aging. Among those, a severe alteration of adult hippocampal neurogenesis was identified as an early event in the etiology of AD in these mice, preceding excitatory and inhibitory networks remodeling in the dentate gyrus. Ultimately, these changes may constitute key components of hippocampal dysfunction and may contribute to the cognitive deficits observed in Tg2576 mice.

Hippocampal anatomical modifications have been reported in several mouse models of AD. Most studies focused on mouse lines mimicking a severe form of the pathology associated with dramatic increase of Aβ production and accelerated onset of amyloidosis, such as APPxPS1dE9 mice [11,19], hAPP-J20 mice [6,8,20] or APPxPS1Ki mice [22]. However, aging being the main risk factor for AD, we used here the well-described Tg2576 mouse model to examine whether the occurrence of hippocampal network alterations relates to typical stages of the disease progression. Indeed, Tg2576 mice are known to develop slow progression of age-dependent amyloidosis and cognitive deficits mimicking the human pathology by several aspects, which allowed us to examine hippocampal damage at ages that correspond to key stages of the pathology.

Recently, several studies have described hAPP/Aβ-dependent modifications in the hippocampus of AD mouse models [8,9,11,38,50]. Among them, variations in the expression of NPY are known to reflect imbalances between excitatory and inhibitory neuronal activities in the hippocampus [51,52]. Here, we explored the level and pattern of NPY expression as a function of age and disease severity in Tg2576 mice. Aberrant NPY expression was not observed in the molecular layer of Tg2576 mice before 18 months of age, extending our recent findings showing intact NPY expression in the molecular layer of 13 month-old Tg2576 females [31]. Expression of NPY in the molecular layer originates from hilar GABAergic interneurons [8], which in normal conditions inhibit the dendrites of granule cells [53]. Therefore, the sprouting of NPY fibers observed in aged Tg2576 mice is likely to enhance granule cells inhibition, which in turn, may participate to cognitive deficits in these mice. However, we observed a subpopulation of younger Tg2576 individuals that displayed ectopic NPY expression in their mossy fibers, which is one of the most frequent pattern of morphological changes described in the rodent hippocampus in association with electrically or chemically induced seizures [39,52,54]. Interestingly, this subpopulation of mice tends to grow with age and disease severity.

Our data also pointed out a decrease in the expression of the activity-related protein CB in the dentate gyrus of Tg2576 mice. A reduction of CB expression was observed in Tg2576 mice granule cells starting at 5 months and worsening with age. Around 6 months of age, insoluble forms of the Aβ peptide become detectable in Tg2576 mouse’s brain, in quantities that increase thereafter [5,26], likely reflecting a transition in the progression of the pathology in this model. Although the link between amyloidogenesis and CB reduction is still unclear, it was reported that reduction of CB levels in the dentate gyrus of hAPP-J20 mice depended on the relative abundance of soluble oligomers but not on the amount of amyloid plaques [6]. CB loss is relevant of functional alteration, as demonstrated by its tight correlation with spatial learning deficits in hAPP-J20 mice [6] and its association with seizure phenotype in APPxPS1 mice [11]. We have previously found that CB levels were not affected in the molecular layer of 13 month-old Tg2576 females [31], suggesting that the progression of the disease might differ between males and females. Sex influence on cerebral amyloid pathology has been described in these mice, but it was reported that 15-19 month-old females were more severely affected than males [55], an argument which does not support our findings.

Hippocampal network rearrangements as evidenced by NPY sprouting and CB depletion in Tg2576 mice are thought to reflect compensatory mechanisms triggered by abnormal network activity. This remodeling became clearly visible at 12 months, an age when Tg2576 mice start forming amyloid plaques in the hippocampus and cortex [23,26] and display well described memory impairments [30]. Whether this remodeling resulted from chronic and long-lasting exposure to abnormal activity only, or whether elevated hippocampal levels of soluble oligomeric Aβ also triggered it, remains to be determined. Supporting the latest possibility, electrophysiological recordings in wild-type brain slices have shown increased excitability and abnormal depolarization in cortical and hippocampal cells when slices were pre-incubated with fibrillar Aβ1-42 peptide [11]. Similar observations were made from slice recordings of pyramidal cells of APPxPS1 transgenic mice [11]. Moreover, *in vivo* studies showed that soluble Aβ including monomers and oligomers also increases the fraction of hyperactive neurons in the hippocampus of AD mouse model [56]. Futhermore, clusters of hyperactive neurons were observed at the vicinity of amyloid plaques [57]. Taken together, these data suggest that abnormal pattern of NPY and CB expression in the hippocampus of Tg2576 mice might reflect compensatory inhibitory response to Aβ-induced aberrant excitatory neuronal activity [58].

We next investigated how these local network rearrangements in Tg2576 mice, may impact adult neurogenesis, a form of hippocampal plasticity involved in memory processes [42,47]. Despite being a widely used model of AD, no extensive report is available regarding adult neurogenesis during aging and disease progression in the Tg2576 mouse line [59,60]. We demonstrated here that hippocampal neurogenesis was quantitatively and qualitatively altered as early as 3 month-old in Tg2576 mice, when these animals mimic a prodromal stage of AD pathology [23]. In the dentate gyrus of young Tg2576 mice, despite high proliferative activity, only few new neurons survived, suggesting that the local environment may be deleterious to young neurons. In Tg2576 mice, it was previously reported that proliferation is decreased in the dentate gyrus of 3-, 6-, and 9 month-old transgenic mice [59]. However, these data based on a 3 days long BrdU injection protocol are likely to reflect short-term survival of BrdU+ cells rather than cell proliferation *per se* [59]. Other studies on the effect of amyloid peptide on cell proliferation in the adult brain have led to conflicting observations likely due to the use of different isoforms and aggregation states of Aβ [61–63]. Our data suggest that elevated levels of soluble oligomers in young Tg2576 mice [3,24,25] stimulated the proliferation of neural progenitors and altered their differentiation and maturation into neurons. More importantly, whilst surviving new neurons in Tg2576 mice achieve normal migration within the granule cell layer, we found that they display shorter dendritic arborization, limited axonal growth and fewer dendritic spines at 28dpi. Whether such altered morphology reflects a delay or a defect in their maturation remains to be determined. However, such conspicuous reduction of their dendritic arbor combined with their limited axonal projection likely impairs new neurons capacity to receive synaptic inputs. Consequently, it might compromise their functional integration into the hippocampal network. Indeed, the specific contribution of adult-born neurons to memory processes relies on their capacity to display highly plastic properties during the critical period of their development (between 14 and 28dpi) [47]. During this time-window, new neurons in the hippocampus of Tg2576 mice showed fewer dendritic spines and shorter axonal projection that likely jeopardize their connectivity and thus, their recruitment into neuronal networks that sustain memory. These morphological alterations might also compromise their ability to survive. Thus, rather than being a side effect of the pathology, altered hippocampal adult-neurogenesis, early in the course of AD progression, may contribute to the alterations of hippocampal-dependent synaptic plasticity and memory processes previously reported in Tg2576 mice [23,64]. Morphological deficits of adult-born granule neurons at late stages of their maturation were also observed in young hAPP-J20 mouse models of AD [65]. However, even though hAPP-J20 animals are free of amyloid plaques at this age, they already display high levels of soluble Aβ as well as aberrant NPY sprouting. Here, using Tg2576 mice, we show that impaired neurogenesis may anticipate these changes in NPY expression.

We found that new neurons morphology was altered prior to amyloid plaque formation in 3 month-old Tg2576 mice. In line with our data, it was shown that mature cortical and hippocampal neurons in Tg2576 mice also displayed dendritic spine loss, prior to plaque deposition [64,66–70]. Furthermore, it was found that modulating Aβ levels using a γ-secretase inhibitor in 3 month-old Tg2576 mice led to the rescue of hippocampal dendritic spines alterations and memory performances [64]. While these findings support the hypothesis that Aβ would be responsible for such changes, directly or indirectly, the possibility that a cell-autonomous effect, driven by hAPP expression itself, contributes to the alteration of hippocampal neurogenesis, cannot be excluded. In line with this idea, the chronic expression of different variants of hAPP in neuronal progenitor cells of the hippocampus, was found to delay dendritic growth, independently of the amyloidogenic capacity of hAPP [71]. Thus, further work should help determine to which extent, abnormal neurogenesis in 3 month-old Tg2576 mice may be related to the presence of hAPP itself or to high levels of soluble Aβ.

Aside from these APP-related processes, other mechanisms may play a part in the early disruption of neurogenesis that we observed in the hippocampus of young Tg2576 mice. Although never explored in Tg2576 mice, a deficit in parvalbumin inhibitory interneuron function [9] or number [72] has been reported in other mouse models of AD. Furthermore, it was recently shown that the release of GABA from hippocampal parvalbumin interneurons maintained adult neural stem cells in a quiescent state, and that specific inactivation of these interneurons led to increased cell proliferation [73]. These observations suggest that similar mechanisms could contribute to the early perturbation of adult neurogenesis seen in Tg2576 mice. It also suggests that some imbalance between GABAergic and glutamatergic transmission might occur before deep anatomical remodeling can be observed.

In summary, Tg2576 mice exhibit a remodeling of their inhibitory and excitatory circuits in the dentate gyrus that takes place along disease progression while altered hippocampal neurogenesis accounts for an early hallmark of the disease in this model. Ultimately, these modifications are likely to contribute to the mechanisms that lead to memory deficits in these mice. Recent advances in the detection of neurogenesis in the living brain may open new avenues for the use of this marker to detect AD in patients at earlier stages [74]. Moreover, our data also indicate that despite altered, adult hippocampal neurogenesis persists throughout age and disease progression, indicating that strategies designed to stimulate neurogenesis *in vivo* may be suitable in the AD brain.

## References

[1] Freir DB, Fedriani R, Scully D, Smith IM, Selkoe DJ et al. (2011) Abeta oligomers inhibit synapse remodelling necessary for memory consolidation. Neurobiol Aging 32: 2211-2218. doi:10.1016/j.neurobiolaging.2010.01.001. PubMed: 20097446. 20097446 10.1016/j.neurobiolaging.2010.01.001 PMC2891223

[2] Lacor PN, Buniel MC, Furlow PW, Clemente AS, Velasco PT et al. (2007) Abeta oligomer-induced aberrations in synapse composition, shape, and density provide a molecular basis for loss of connectivity in Alzheimer’s disease. J Neurosci 27: 796-807. doi:10.1523/JNEUROSCI.3501-06.2007. PubMed: 17251419. 17251419 10.1523/JNEUROSCI.3501-06.2007 PMC6672917

[3] Lesné S, Koh MT, Kotilinek L, Kayed R, Glabe CG et al. (2006) A specific amyloid-beta protein assembly in the brain impairs memory. Nature 440: 352-357. doi:10.1038/nature04533. PubMed: 16541076. 16541076 10.1038/nature04533

[4] Rabinovici GD, Jagust WJ (2009) Amyloid imaging in aging and dementia: testing the amyloid hypothesis in vivo. Behav Neurol 21: 117-128. PubMed: 19847050. 19847050 10.3233/BEN-2009-0232 PMC2804478

[5] Westerman MA, Cooper-Blacketer D, Mariash A, Kotilinek L, Kawarabayashi T et al. (2002) The relationship between Abeta and memory in the Tg2576 mouse model of Alzheimer’s disease. J Neurosci 22: 1858-1867. PubMed: 11880515. 11880515 10.1523/JNEUROSCI.22-05-01858.2002 PMC6758862

[6] Palop JJ, Jones B, Kekonius L, Chin J, Yu GQ et al. (2003) Neuronal depletion of calcium-dependent proteins in the dentate gyrus is tightly linked to Alzheimer’s disease-related cognitive deficits. Proc Natl Acad Sci U S A 100: 9572-9577. doi:10.1073/pnas.1133381100. PubMed: 12881482. 12881482 10.1073/pnas.1133381100 PMC170959

[7] Palop JJ, Chin J, Bien-Ly N, Massaro C, Yeung BZ et al. (2005) Vulnerability of dentate granule cells to disruption of arc expression in human amyloid precursor protein transgenic mice. J Neurosci 25: 9686-9693. doi:10.1523/JNEUROSCI.2829-05.2005. PubMed: 16237173. 16237173 10.1523/JNEUROSCI.2829-05.2005 PMC6725729

[8] Palop JJ, Chin J, Roberson ED, Wang J, Thwin MT et al. (2007) Aberrant excitatory neuronal activity and compensatory remodeling of inhibitory hippocampal circuits in mouse models of Alzheimer’s disease. Neuron 55: 697-711. doi:10.1016/j.neuron.2007.07.025. PubMed: 17785178. 17785178 10.1016/j.neuron.2007.07.025 PMC8055171

[9] Verret L, Mann EO, Hang GB, Barth AM, Cobos I et al. (2012) Inhibitory interneuron deficit links altered network activity and cognitive dysfunction in Alzheimer model. Cell 149: 708-721. doi:10.1016/j.cell.2012.02.046. PubMed: 22541439. 22541439 10.1016/j.cell.2012.02.046 PMC3375906

[10] Chin J, Palop JJ, Puoliväli J, Massaro C, Bien-Ly N et al. (2005) Fyn kinase induces synaptic and cognitive impairments in a transgenic mouse model of Alzheimer’s disease. J Neurosci 25: 9694-9703. doi:10.1523/JNEUROSCI.2980-05.2005. PubMed: 16237174. 16237174 10.1523/JNEUROSCI.2980-05.2005 PMC6725734

[11] Minkeviciene R, Rheims S, Dobszay MB, Zilberter M, Hartikainen J et al. (2009) Amyloid beta-induced neuronal hyperexcitability triggers progressive epilepsy. J Neurosci 29: 3453-3462. doi:10.1523/JNEUROSCI.5215-08.2009. PubMed: 19295151. 19295151 10.1523/JNEUROSCI.5215-08.2009 PMC6665248

[12] Palop JJ, Mucke L (2010) Synaptic depression and aberrant excitatory network activity in Alzheimer’s disease: two faces of the same coin? Neuromol Med 12: 48-55. doi:10.1007/s12017-009-8097-7. PubMed: 19838821. 10.1007/s12017-009-8097-7 PMC3319077 19838821

[13] Lazarov O, Marr RA (2010) Neurogenesis and Alzheimer’s disease: at the crossroads. Exp Neurol 223: 267-281. doi:10.1016/j.expneurol.2009.08.009. PubMed: 19699201. 19699201 10.1016/j.expneurol.2009.08.009 PMC2864344

[14] Verret L, Trouche S, Zerwas M, Rampon C (2007) Hippocampal neurogenesis during normal and pathological aging. Psychoneuroendocrinology 32 Suppl 1: S26-S30. doi:10.1016/j.psyneuen.2007.04.014. PubMed: 17629417. 17629417 10.1016/j.psyneuen.2007.04.014

[15] Trouche S, Bontempi B, Roullet P, Rampon C (2009) Recruitment of adult-generated neurons into functional hippocampal networks contributes to updating and strengthening of spatial memory. Proc Natl Acad Sci U S A 106: 5919-5924. doi:10.1073/pnas.0811054106. PubMed: 19321751. 19321751 10.1073/pnas.0811054106 PMC2667087

[16] Zhao C, Deng W, Gage FH (2008) Mechanisms and functional implications of adult neurogenesis. Cell 132: 645-660. doi:10.1016/j.cell.2008.01.033. PubMed: 18295581. 18295581 10.1016/j.cell.2008.01.033

[17] Lazarov O, Demars MP (2012) All in the Family: How the APPs Regulate Neurogenesis. Front Neurosci 6: 81 PubMed: 22675290. 22675290 10.3389/fnins.2012.00081 PMC3366480

[18] Popović M, Caballero-Bleda M, Kadish I, Van Groen T (2008) Subfield and layer-specific depletion in calbindin-D28K, calretinin and parvalbumin immunoreactivity in the dentate gyrus of amyloid precursor protein/presenilin 1 transgenic mice. Neuroscience 155: 182-191. doi:10.1016/j.neuroscience.2008.05.023. PubMed: 18583063. 18583063 10.1016/j.neuroscience.2008.05.023 PMC2596990

[19] Verret L, Jankowsky JL, Xu GM, Borchelt DR, Rampon C (2007) Alzheimer’s-type amyloidosis in transgenic mice impairs survival of newborn neurons derived from adult hippocampal neurogenesis. J Neurosci 27: 6771-6780. doi:10.1523/JNEUROSCI.5564-06.2007. PubMed: 17581964. 17581964 10.1523/JNEUROSCI.5564-06.2007 PMC4439193

[20] Sun B, Halabisky B, Zhou Y, Palop JJ, Yu G et al. (2009) Imbalance between GABAergic and Glutamatergic Transmission Impairs Adult Neurogenesis in an Animal Model of Alzheimer’s Disease. Cell Stem Cell 5: 624-633. doi:10.1016/j.stem.2009.10.003. PubMed: 19951690. 19951690 10.1016/j.stem.2009.10.003 PMC2823799

[21] Cotel MC, Bayer TA, Wirths O (2008) Age-dependent loss of dentate gyrus granule cells in APP/PS1KI mice. Brain Res 1222: 207-213. doi:10.1016/j.brainres.2008.05.052. PubMed: 18585693. 18585693 10.1016/j.brainres.2008.05.052

[22] Faure A, Verret L, Bozon B, El Tannir El Tayara N, Ly M et al. (2011) Impaired neurogenesis, neuronal loss, and brain functional deficits in the APPxPS1-Ki mouse model of Alzheimer’s disease. Neurobiol Aging 32: 407-418. doi:10.1016/j.neurobiolaging.2009.03.009. PubMed: 19398247. 19398247 10.1016/j.neurobiolaging.2009.03.009

[23] Hsiao K, Chapman P, Nilsen S, Eckman C, Harigaya Y et al. (1996) Correlative memory deficits, Abeta elevation, and amyloid plaques in transgenic mice. Science 274: 99-102. doi:10.1126/science.274.5284.99. PubMed: 8810256. 8810256 10.1126/science.274.5284.99

[24] Klingner M, Apelt J, Kumar A, Sorger D, Sabri O et al. (2003) Alterations in cholinergic and non-cholinergic neurotransmitter receptor densities in transgenic Tg2576 mouse brain with beta-amyloid plaque pathology. Int J Dev Neurosci 21: 357-369. doi:10.1016/j.ijdevneu.2003.08.001. PubMed: 14599482. 14599482 10.1016/j.ijdevneu.2003.08.001

[25] Unger C, Hedberg MM, Mustafiz T, Svedberg MM, Nordberg A (2005) Early changes in Abeta levels in the brain of APPswe transgenic mice--implication on synaptic density, alpha7 neuronal nicotinic acetylcholine- and N-methyl-D-aspartate receptor levels. Mol Cell Neurosci 30: 218-227. doi:10.1016/j.mcn.2005.07.012. PubMed: 16107318. 16107318 10.1016/j.mcn.2005.07.012

[26] Kawarabayashi T, Younkin LH, Saido TC, Shoji M, Ashe KH et al. (2001) Age-dependent changes in brain, CSF, and plasma amyloid (beta) protein in the Tg2576 transgenic mouse model of Alzheimer’s disease. J Neurosci 21: 372-381. PubMed: 11160418. 11160418 10.1523/JNEUROSCI.21-02-00372.2001 PMC6763819

[27] Arendash GW, King DL (2002) Intra- and intertask relationships in a behavioral test battery given to Tg2576 transgenic mice and controls. Physiol Behav 75: 643-652. doi:10.1016/S0031-9384(02)00640-6. PubMed: 12020729. 12020729 10.1016/s0031-9384(02)00640-6

[28] Lassalle JM, Halley H, Daumas S, Verret L, Francés B (2008) Effects of the genetic background on cognitive performances of TG2576 mice. Behav Brain Res 191: 104-110. doi:10.1016/j.bbr.2008.03.017. PubMed: 18433892. 18433892 10.1016/j.bbr.2008.03.017

[29] Mitchell JC, Ariff BB, Yates DM, Lau KF, Perkinton MS et al. (2009) X11beta rescues memory and long-term potentiation deficits in Alzheimer’s disease APPswe Tg2576 mice. Hum Mol Genet 18: 4492-4500. doi:10.1093/hmg/ddp408. PubMed: 19744962. 19744962 10.1093/hmg/ddp408 PMC2773264

[30] Stewart S, Cacucci F, Lever C (2011) Which memory task for my mouse? A systematic review of spatial memory performance in the Tg2576 Alzheimer’s mouse model. J Alzheimers Dis 26: 105-126. 10.3233/JAD-2011-101827 21558645

[31] Verret L, Krezymon A, Halley H, Trouche S, Zerwas M et al. (2013) Transient enriched housing before amyloidosis onset sustains cognitive improvement in Tg2576 mice. Neurobiol Aging, 34: 211–25. PubMed: 22727275. 22727275 10.1016/j.neurobiolaging.2012.05.013

[32] Hsiao KK, Borchelt DR, Olson K, Johannsdottir R, Kitt C et al. (1995) Age-related CNS disorder and early death in transgenic FVB/N mice overexpressing Alzheimer amyloid precursor proteins. Neuron 15: 1203-1218. doi:10.1016/0896-6273(95)90107-8. PubMed: 7576662. 7576662 10.1016/0896-6273(95)90107-8

[33] Roybon L, Hjalt T, Stott S, Guillemot F, Li JY et al. (2009) Neurogenin2 directs granule neuroblast production and amplification while NeuroD1 specifies neuronal fate during hippocampal neurogenesis. PLOS ONE 4: e4779. doi:10.1371/journal.pone.0004779. PubMed: 19274100. 19274100 10.1371/journal.pone.0004779 PMC2652712

[34] Espósito MS, Piatti VC, Laplagne DA, Morgenstern NA, Ferrari CC et al. (2005) Neuronal differentiation in the adult hippocampus recapitulates embryonic development. J Neurosci 25: 10074-10086. doi:10.1523/JNEUROSCI.3114-05.2005. PubMed: 16267214. 16267214 10.1523/JNEUROSCI.3114-05.2005 PMC6725804

[35] Zhao C, Teng EM, Summers RG Jr., Ming GL, Gage FH (2006) Distinct morphological stages of dentate granule neuron maturation in the adult mouse hippocampus. J Neurosci 26: 3-11. doi:10.1523/JNEUROSCI.3648-05.2006. PubMed: 16399667. 16399667 10.1523/JNEUROSCI.3648-05.2006 PMC6674324

[36] Harris KM, Jensen FE, Tsao B (1992) Three-dimensional structure of dendritic spines and synapses in rat hippocampus (CA1) at postnatal day 15 and adult ages: implications for the maturation of synaptic physiology and long-term potentiation. J Neurosci 12: 2685-2705. PubMed: 1613552. 1613552 10.1523/JNEUROSCI.12-07-02685.1992 PMC6575840

[37] Palop JJ, Mucke L (2009) Epilepsy and cognitive impairments in Alzheimer disease. Arch Neurol 66: 435-440. doi:10.1001/archneurol.2009.15. PubMed: 19204149. 19204149 10.1001/archneurol.2009.15 PMC2812914

[38] Harris JA, Devidze N, Verret L, Ho K, Halabisky B et al. (2010) Transsynaptic progression of amyloid-beta-induced neuronal dysfunction within the entorhinal-hippocampal network. Neuron 68: 428-441. doi:10.1016/j.neuron.2010.10.020. PubMed: 21040845. 21040845 10.1016/j.neuron.2010.10.020 PMC3050043

[39] Sperk G, Marksteiner J, Gruber B, Bellmann R, Mahata M et al. (1992) Functional changes in neuropeptide Y- and somatostatin-containing neurons induced by limbic seizures in the rat. Neuroscience 50: 831-846. doi:10.1016/0306-4522(92)90207-I. PubMed: 1360155. 1360155 10.1016/0306-4522(92)90207-i

[40] Cheng IH, Scearce-Levie K, Legleiter J, Palop JJ, Gerstein H et al. (2007) Accelerating amyloid-beta fibrillization reduces oligomer levels and functional deficits in Alzheimer disease mouse models. J Biol Chem 282: 23818-23828. doi:10.1074/jbc.M701078200. PubMed: 17548355. 17548355 10.1074/jbc.M701078200

[41] Dickey CA, Loring JF, Montgomery J, Gordon MN, Eastman PS et al. (2003) Selectively reduced expression of synaptic plasticity-related genes in amyloid precursor protein + presenilin-1 transgenic mice. J Neurosci 23: 5219-5226. PubMed: 12832546. 12832546 10.1523/JNEUROSCI.23-12-05219.2003 PMC6741153

[42] Marín-Burgin A, Schinder AF (2012) Requirement of adult-born neurons for hippocampus-dependent learning. Behav Brain Res 227: 391-399. doi:10.1016/j.bbr.2011.07.001. PubMed: 21763727. 21763727 10.1016/j.bbr.2011.07.001

[43] Song J, Christian KM, Ming GL, Song H (2012) Modification of hippocampal circuitry by adult neurogenesis. Dev Neurobiol 72: 1032-1043. doi:10.1002/dneu.22014. PubMed: 22354697. 22354697 10.1002/dneu.22014 PMC3710549

[44] Kempermann G, Gast D, Kronenberg G, Yamaguchi M, Gage FH (2003) Early determination and long-term persistence of adult-generated new neurons in the hippocampus of mice. Development 130: 391-399. doi:10.1242/dev.00203. PubMed: 12466205. 12466205 10.1242/dev.00203

[45] Couillard-Despres S, Winner B, Schaubeck S, Aigner R, Vroemen M et al. (2005) Doublecortin expression levels in adult brain reflect neurogenesis. Eur J Neurosci 21: 1-14. doi:10.1111/j.1460-9568.2004.03813.x. PubMed: 15654838. 15654838 10.1111/j.1460-9568.2004.03813.x

[46] Pathania M, Yan LD, Bordey A (2010) A symphony of signals conducts early and late stages of adult neurogenesis. Neuropharmacology 58: 865-876. doi:10.1016/j.neuropharm.2010.01.010. PubMed: 20097213. 20097213 10.1016/j.neuropharm.2010.01.010 PMC2850602

[47] Deng W, Aimone JB, Gage FH (2010) New neurons and new memories: how does adult hippocampal neurogenesis affect learning and memory? Nat Rev Neurosci 11: 339-350. doi:10.1038/nrn2822. PubMed: 20354534. 20354534 10.1038/nrn2822 PMC2886712

[48] Nimchinsky EA, Sabatini BL, Svoboda K (2002) Structure and function of dendritic spines. Annu Rev Physiol 64: 313-353. doi:10.1146/annurev.physiol.64.081501.160008. PubMed: 11826272. 11826272 10.1146/annurev.physiol.64.081501.160008

[49] Sala C (2002) Molecular regulation of dendritic spine shape and function. Neurosignals 11: 213-223. doi:10.1159/000065433. PubMed: 12393947. 12393947 10.1159/000065433

[50] Roberson ED, Halabisky B, Yoo JW, Yao J, Chin J et al. (2011) Amyloid-beta/Fyn-induced synaptic, network, and cognitive impairments depend on tau levels in multiple mouse models of Alzheimer’s disease. J Neurosci 31: 700-711. doi:10.1523/JNEUROSCI.4152-10.2011. PubMed: 21228179. 21228179 10.1523/JNEUROSCI.4152-10.2011 PMC3325794

[51] Sperk G, Hamilton T, Colmers WF (2007) Neuropeptide Y in the dentate gyrus. Prog Brain Res 163: 285-297. doi:10.1016/S0079-6123(07)63017-9. PubMed: 17765725. 17765725 10.1016/S0079-6123(07)63017-9

[52] Vezzani A, Sperk G, Colmers WF (1999) Neuropeptide Y: emerging evidence for a functional role in seizure modulation. Trends Neurosci 22: 25-30. doi:10.1016/S0166-2236(98)01284-3. PubMed: 10088996. 10088996 10.1016/s0166-2236(98)01284-3

[53] Freund TF, Buzsáki G (1996) Interneurons of the hippocampus. Hippocampus 6: 347-470. PubMed: 8915675. 8915675 10.1002/(SICI)1098-1063(1996)6:4<347::AID-HIPO1>3.0.CO;2-I

[54] Scharfman HE, Gray WP (2006) Plasticity of neuropeptide Y in the dentate gyrus after seizures, and its relevance to seizure-induced neurogenesis. EXS: 193-211. PubMed: 16383008. 16383008 10.1007/3-7643-7417-9_15 PMC4398306

[55] Callahan MJ, Lipinski WJ, Bian F, Durham RA, Pack A et al. (2001) Augmented senile plaque load in aged female beta-amyloid precursor protein-transgenic mice. Am J Pathol 158: 1173-1177. doi:10.1016/S0002-9440(10)64064-3. PubMed: 11238065. 11238065 10.1016/s0002-9440(10)64064-3 PMC1850367

[56] Busche MA, Chen X, Henning HA, Reichwald J, Staufenbiel M et al. (2012) Critical role of soluble amyloid-beta for early hippocampal hyperactivity in a mouse model of Alzheimer’s disease. Proc Natl Acad Sci U S A 109: 8740-8745. doi:10.1073/pnas.1206171109. PubMed: 22592800. 22592800 10.1073/pnas.1206171109 PMC3365221

[57] Busche MA, Eichhoff G, Adelsberger H, Abramowski D, Wiederhold KH et al. (2008) Clusters of hyperactive neurons near amyloid plaques in a mouse model of Alzheimer’s disease. Science 321: 1686-1689. doi:10.1126/science.1162844. PubMed: 18802001. 18802001 10.1126/science.1162844

[58] Palop JJ, Mucke L (2010) Amyloid-beta-induced neuronal dysfunction in Alzheimer’s disease: from synapses toward neural networks. Nat Neurosci 13: 812-818. doi:10.1038/nn.2583. PubMed: 20581818. 20581818 10.1038/nn.2583 PMC3072750

[59] Dong H, Goico B, Martin M, Csernansky CA, Bertchume A et al. (2004) Modulation of hippocampal cell proliferation, memory, and amyloid plaque deposition in APPsw (Tg2576) mutant mice by isolation stress. Neuroscience 127: 601-609. doi:10.1016/j.neuroscience.2004.05.040. PubMed: 15283960. 15283960 10.1016/j.neuroscience.2004.05.040

[60] Ihunwo AO, Schliebs R (2010) Cell proliferation and total granule cell number in dentate gyrus of transgenic Tg2576 mouse. Acta Neurobiol Exp Wars 70: 362-369. PubMed: 21196944. 21196944 10.55782/ane-2010-1808

[61] Haughey NJ, Nath A, Chan SL, Borchard AC, Rao MS et al. (2002) Disruption of neurogenesis by amyloid beta-peptide, and perturbed neural progenitor cell homeostasis, in models of Alzheimer’s disease. J Neurochem 83: 1509-1524. doi:10.1046/j.1471-4159.2002.01267.x. PubMed: 12472904. 12472904 10.1046/j.1471-4159.2002.01267.x

[62] He P, Shen Y (2009) Interruption of beta-catenin signaling reduces neurogenesis in Alzheimer’s disease. J Neurosci 29: 6545-6557. doi:10.1523/JNEUROSCI.0421-09.2009. PubMed: 19458225. 19458225 10.1523/JNEUROSCI.0421-09.2009 PMC3618977

[63] López-Toledano MA, Shelanski ML (2004) Neurogenic effect of beta-amyloid peptide in the development of neural stem cells. J Neurosci 24: 5439-5444. doi:10.1523/JNEUROSCI.0974-04.2004. PubMed: 15190117. 15190117 10.1523/JNEUROSCI.0974-04.2004 PMC6729298

[64] D’Amelio M, Cavallucci V, Middei S, Marchetti C, Pacioni S et al. (2011) Caspase-3 triggers early synaptic dysfunction in a mouse model of Alzheimer’s disease. Nat Neurosci 14: 69-76. doi:10.1038/nn.2709. PubMed: 21151119. 21151119 10.1038/nn.2709

[65] Sun B, Halabisky B, Zhou Y, Palop JJ, Yu G et al. (2009) Imbalance between GABAergic and Glutamatergic Transmission Impairs Adult Neurogenesis in an Animal Model of Alzheimer’s Disease. Cell Stem Cell Volumes 6: 10-633 1016/j.stem.2009.10.003 10.1016/j.stem.2009.10.003 PMC2823799 19951690

[66] Adlard PA, Bica L, White AR, Nurjono M, Filiz G et al. (2011) Metal ionophore treatment restores dendritic spine density and synaptic protein levels in a mouse model of Alzheimer’s disease. PLOS ONE 6: e17669. doi:10.1371/journal.pone.0017669. PubMed: 21412423. 21412423 10.1371/journal.pone.0017669 PMC3055881

[67] Alpár A, Ueberham U, Brückner MK, Seeger G, Arendt T et al. (2006) Different dendrite and dendritic spine alterations in basal and apical arbors in mutant human amyloid precursor protein transgenic mice. Brain Res 1099: 189-198. doi:10.1016/j.brainres.2006.04.109. PubMed: 16781686. 16781686 10.1016/j.brainres.2006.04.109

[68] Jacobsen JS, Wu CC, Redwine JM, Comery TA, Arias R et al. (2006) Early-onset behavioral and synaptic deficits in a mouse model of Alzheimer’s disease. Proc Natl Acad Sci U S A 103: 5161-5166. doi:10.1073/pnas.0600948103. PubMed: 16549764. 16549764 10.1073/pnas.0600948103 PMC1405622

[69] Lanz TA, Carter DB, Merchant KM (2003) Dendritic spine loss in the hippocampus of young PDAPP and Tg2576 mice and its prevention by the ApoE2 genotype. Neurobiol Dis 13: 246-253. doi:10.1016/S0969-9961(03)00079-2. PubMed: 12901839. 12901839 10.1016/s0969-9961(03)00079-2

[70] Perez-Cruz C, Nolte MW, van Gaalen MM, Rustay NR, Termont A et al. (2011) Reduced spine density in specific regions of CA1 pyramidal neurons in two transgenic mouse models of Alzheimer’s disease. J Neurosci 31: 3926-3934. doi:10.1523/JNEUROSCI.6142-10.2011. PubMed: 21389247. 21389247 10.1523/JNEUROSCI.6142-10.2011 PMC6622797

[71] Morgenstern NA, Giacomini D, Lombardi G, Castaño EM, Schinder AF (2013) Delayed dendritic development in newly generated dentate granule cells by cell-autonomous expression of the amyloid precursor protein. Mol Cell Neurosci 56: 298–306. doi:10.1016/j.mcn.2013.07.003. PubMed: 23851186. 23851186 10.1016/j.mcn.2013.07.003 PMC3791211

[72] Takahashi H, Brasnjevic I, Rutten BP, Van Der Kolk N, Perl DP et al. (2010) Hippocampal interneuron loss in an APP/PS1 double mutant mouse and in Alzheimer’s disease. Brain Struct Funct 214: 145-160. doi:10.1007/s00429-010-0242-4. PubMed: 20213270. 20213270 10.1007/s00429-010-0242-4 PMC3038332

[73] Song J, Zhong C, Bonaguidi MA, Sun GJ, Hsu D et al. (2012) Neuronal circuitry mechanism regulating adult quiescent neural stem-cell fate decision. Nature 489: 150-154. doi:10.1038/nature11306. PubMed: 22842902. 22842902 10.1038/nature11306 PMC3438284

[74] Couillard-Despres S, Iglseder B, Aigner L (2011) Neurogenesis, cellular plasticity and cognition: the impact of stem cells in the adult and aging brain- a mini-review. Gerontology 57: 559-564. doi:10.1159/000323481. PubMed: 21311170. 21311170 10.1159/000323481

